# Intravascular Large B-Cell Lymphoma Presenting with Diffuse Gallbladder Wall Thickening: A Case Report and Literature Review

**DOI:** 10.1155/2018/2494207

**Published:** 2018-04-22

**Authors:** Sayf Al-Katib, Robert Colvin, Farnoosh Sokhandon

**Affiliations:** Department of Diagnostic Radiology and Molecular Imaging, Oakland University William Beaumont School of Medicine, Beaumont Health, Royal Oak, MI, USA

## Abstract

Intravascular large B-cell lymphoma is a rare subtype of extranodal diffuse B-cell lymphoma characterized by proliferation of neoplastic cells within the lumen of small and medium sized vessels. Clinical and imaging findings are nebulous as the intravascular subtype of lymphoma can involve a multitude of organs. Involvement of the gallbladder is extremely uncommon, and imaging findings can be easily confused for more prevalent pathologies. We report a case of intravascular large B-cell lymphoma in an 83-year-old male and review clinical presentation and imaging findings on CT, ultrasound, hepatobiliary iminodiacetic acid (HIDA) scan, and MRI. It is important for the radiologist to know about this disease as the imaging findings are atypical of other types of lymphoma, and this may lead to a delay in diagnosis and treatment.

## 1. Introduction

Intravascular large B-cell lymphoma is a rare subtype of extranodal diffuse B-cell lymphoma characterized by proliferation of neoplastic cells within the lumen of small and medium sized vessels. Clinical and imaging findings are nebulous as the intravascular subtype of lymphoma can involve a multitude of organs. Involvement of the gallbladder is extremely uncommon, and imaging findings can be easily confused for more prevalent pathologies. This case is a reminder of a rare but important diagnostic consideration in the setting of a diffusely thickened gallbladder wall.

## 2. Case Presentation

An 83-year-old Caucasian male with past medical history of hypertension, diabetes, and coronary artery disease presented to the emergency department with extreme fatigue, weakness, and disorientation. He reported a history of 10–15-pound weight loss over the previous month. Physical exam did not yield any focal signs. Laboratory evaluation revealed pancytopenia (Hgb 9.6 g/dL, WBC 3.0 bil/L, and platelets 169 bil/L) and increased liver function enzymes (AST 311 U/L, ALT 62 U/L, and ALP 237 U/L). The patient was admitted for generalized weakness and fatigue.

**Figure 1:**
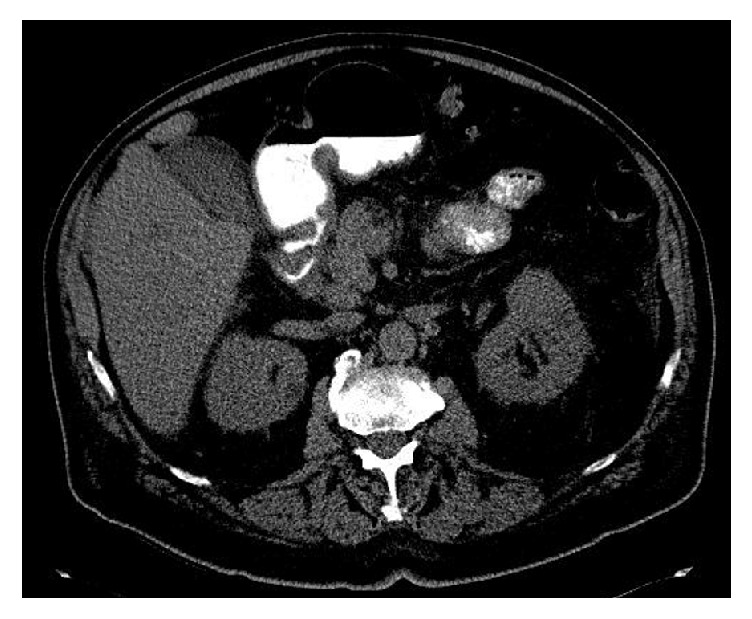
Axial unenhanced CT of the abdomen obtained at presentation shows a diffusely thickened and low attenuation gallbladder wall.

An unenhanced CT scan of the abdomen and pelvis obtained in the emergency department showed a nondistended gallbladder with diffuse wall thickening (Figure 1). A small calcified gallstone was identified in the neck of the gallbladder. Neither a soft tissue mass nor lymphadenopathy was present. Although acute cholecystitis was not a leading differential diagnosis based on the clinical presentation, HIDA scan was recommended based on the unexpected CT findings.

The patient developed worsening alteration of mental status, metabolic acidosis, further increase in liver function tests, and pancytopenia. D-dimer was elevated (1830 ng/mL). There was initially clinical concern for pulmonary embolism versus disseminated intravascular coagulation. Nuclear medicine testing with ventilation-perfusion (VQ) scan was performed and low probability for pulmonary embolism was found. Coagulation testing remained stable.

**Figure 2:**
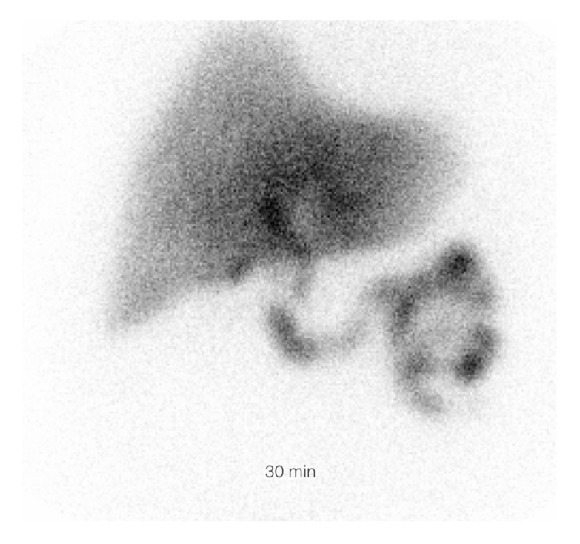
Image obtained during HIDA scan shows early filling of the gallbladder by 30 minutes which was confirmed on delayed 40- and 60-minute images.

**Figure 3:**
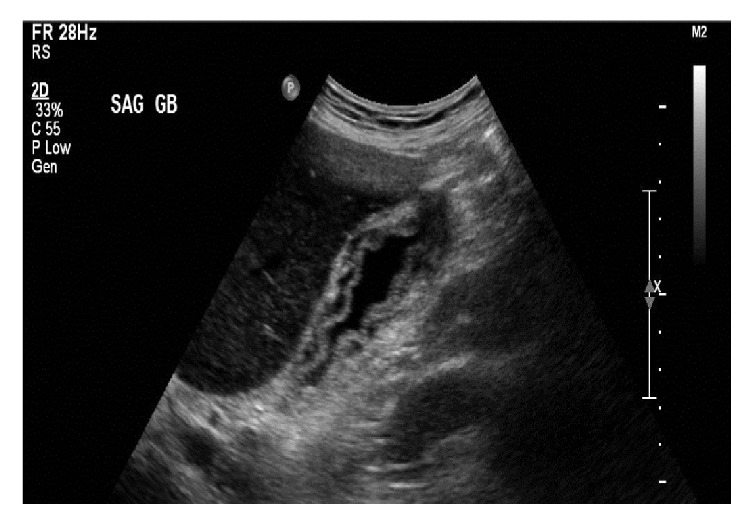
Grayscale longitudinal sonographic image of the right upper quadrant shows a decompressed gallbladder wall with diffuse wall thickening. The gallbladder wall has a striated appearance without increased vascularity.

**Figure 4:**
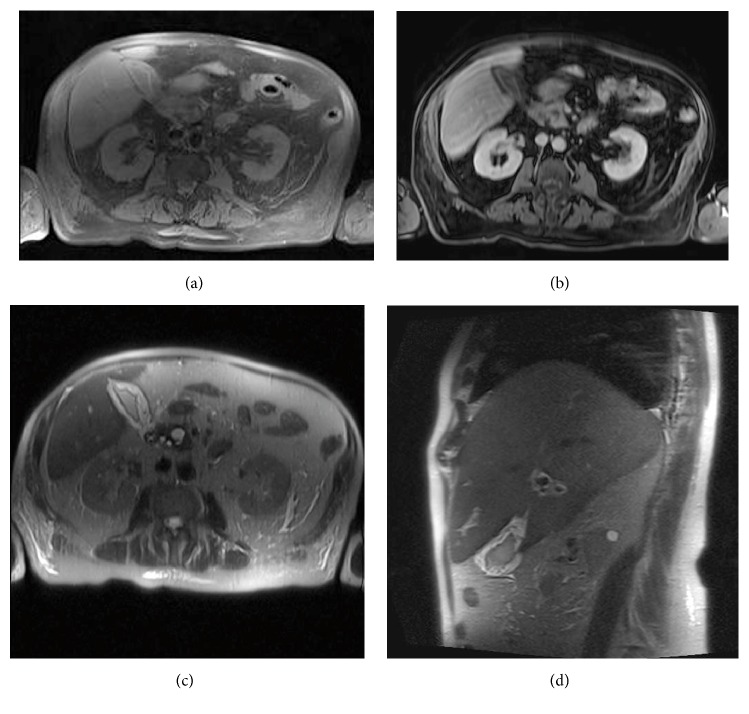
(a) Axial T1W-VIBE precontrast, (b) axial T1W-VIBE postcontrast, (c) axial T2W-fat suppressed, and (d) coronal T2W-fat suppressed sequences. MR images show diffuse gallbladder wall thickening with increased signal intensity on T2 weighted fat suppressed images. Postcontrast fat suppressed images demonstrate homogeneous mucosal enhancement with no transmural wall enhancement.

The HIDA scan revealed cystic duct patency (Figure 2). Subsequently, a targeted sonographic examination of the right upper quadrant was performed to further characterize the nonspecific findings on the CT scan. This revealed diffuse gallbladder wall thickening measuring up to 8 mm. The gallbladder wall had a striated appearance with a central hypoechoic stripe surrounded by an echogenic band on either side (Figure 3). The sonographic Murphy's sign was negative. The gallbladder was decompressed and contained a few small stones. An MRI of the abdomen with contrast showed diffusely thickened gallbladder wall measuring up to 12 mm with intramural increased signal intensity on T2 weighted images. Contrast enhanced T1 weighted sequences showed no abnormal enhancement or nodularity of the gallbladder wall. The gallbladder did contain a few small stones, but there was no luminal distention, pericholecystic fluid, or lymphadenopathy (Figure 4). Clinical concern further developed for septicemia with the gallbladder as the primary site of origin given the imaging findings. The patient underwent laparoscopic cholecystectomy.

**Figure 5:**
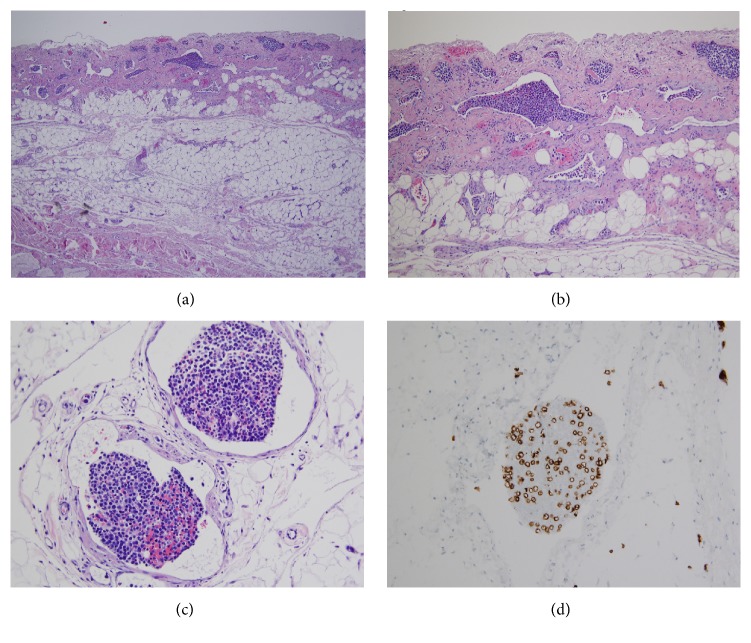
Microscopic images show gallbladder wall thickening and lymphatic ectasia. Several blood vessels in the subserosal fat are infiltrated with large, atypical lymphomatous cells. The mucosa is uninvolved. Immunohistochemical stain with CD79a confirms B-cell origin.

Pathologic evaluation of the surgical specimen revealed findings of intravascular large B-cell lymphoma (IVLBCL), a rare variant of extranodal diffuse large B-cell lymphoma. Histologic evaluation with hematoxylin and eosin stain demonstrated diffuse gallbladder wall thickening with lymphatic ectasia (Figure 5). Several blood vessels in the subserosal fat showed infiltration with large, atypical lymphomatous cells. The mucosa was uninvolved. These features explained all the imaging findings well in this particular case.

Flow cytometry of the peripheral blood revealed a small population of aberrant B-cells which correlate to the pathologic gallbladder wall findings. Subsequent bone marrow core biopsy and aspiration were performed. Pathologic evaluation demonstrated slight hypercellularity of the marrow and 10–15% involvement by diffuse large B-cell lymphoma. Immunohistochemical stain of bone marrow core biopsy sample was positive for CD79a, confirming B-cell origin.

Postoperatively, the patient's condition continued to deteriorate requiring respiratory support in the intensive care unit. He developed severe metabolic acidosis and hemodynamic instability. Given the patient's poor functional status and concomitant diagnosis of IVLBCL, the decision to withdraw supportive care was made by his family. The patient expired 7 days after initial presentation.

## 3. Discussion

Intravascular B-cell lymphoma is a highly aggressive form of extranodal B-cell lymphoma characterized by neoplastic cell growth within the lumen of small sized vessels [1]. It presents as a disseminated aggressive disease without focal mass or lymphadenopathy [2]. Neoplastic vascular involvement can affect all types of organs, such as bone marrow, CNS, skin, lung, adrenal gland, liver, kidney, spleen, thyroid, pituitary gland, and gastrointestinal tract [3]. In our patient, no additional lesions were identified in the base of the lungs, abdomen, or pelvis. No cross-sectional imaging was performed on the chest or brain during this hospitalization, so involvement of the lungs or brain cannot be excluded. Overall, findings in this case are consistent with secondary IVLBCL of the gallbladder wall. IVLBCL presenting with gallbladder symptoms is very rare with only a handful of cases described in the literature [4]. Common clinical presentation includes nonspecific systemic symptoms, such as fever of unknown origin, fatigue, deterioration of performance status, and neurological alteration.

This case highlights the diagnostic conundrum of IVLBCL. In our case, the patient experienced nonspecific intermittent altered mental status and fatigue without focal abdominal pain. Furthermore, imaging evaluation revealed only nonspecific findings of striated gallbladder wall thickening. The lack of lymphadenopathy in patients with IVLBCL also makes timely diagnosis challenging. Additionally, staging of this disease is difficult secondary to the lack of discrete mass.

To our knowledge, the multimodal imaging characteristics of IVLBCL affecting the gallbladder have not been described in the literature. Diffuse gallbladder wall thickening is nonspecific. It can be secondary to primary gallbladder pathology such as acute and chronic cholecystitis, adenomyomatosis, or gallbladder carcinoma [5]. It may also be a manifestation of a host of conditions extrinsic to the gallbladder such as hypoalbuminemia, congestive heart failure, hepatitis, renal failure, and physiologic contraction after a recent meal. A thickened gallbladder wall with a striated sonographic appearance (thick echogenic wall with a homogenous sonolucent central band) is more characteristic of benign noninflammatory conditions [6].

MRI evaluation is helpful in characterizing gallbladder wall thickening as those cases which show early and prolonged enhancement tend to be malignant whereas benign etiologies generally have less rapid and prolonged enhancement [7]. However, our case did not follow this concept. With vessel occlusion by intravascular neoplastic cells, one would expect to find decreased postcontrast enhancement as seen in our case. The lack of a focal mass, lymphadenopathy, and postcontrast enhancement make for an unusual imaging constellation for lymphoma [8]. While findings of neurologic alteration, pancytopenia, and elevated liver function studies have been described in cases of IVLBCL, they are not specific [3]. Additional potentially useful imaging, not obtained in this case, includes diffusion weighted imaging and positron emission tomography (PET). Diffusion weight imaging (DWI) would likely show abnormally restricted diffusion due to the presence of high grade malignant cells. PET would likely show increased FDG activity as with large B-cell lymphoma elsewhere. Unfortunately, these findings overlap with acute cholecystitis and would not help in distinguishing lymphoma from acute inflammation.

IVLBCL is a highly aggressive neoplastic proliferation of B-cells within medium and small sized vessels. The clinical and laboratory findings are not specific, and the diagnosis is often delayed. Furthermore, the imaging findings in our case highlight the diagnostic challenge of IVLBCL. The lack of a focal mass, lymphadenopathy, and abnormal contrast enhancement misleadingly point to a nonneoplastic process. Furthermore, diffuse gallbladder wall thickening with a trilaminar appearance is most characteristic of benign noninflammatory conditions, which would not prompt tissue diagnosis. A benign condition such as diffuse adenomyomatosis with numerous Rokitansky-Aschoff sinuses could be considered, but this case had no cystic changes and the trilaminar appearance of the gallbladder would be an unusual imaging finding. Although no diagnostic imaging features of IVLBCL exist, with increased awareness, the antemortem identification of this elusive diagnosis may be suggested.
